# Prevalence and Risk Factors for Unruptured Intracranial Aneurysms in the Population at High Risk for Aneurysm in the Rural Areas of Tianjin

**DOI:** 10.3389/fneur.2022.853054

**Published:** 2022-03-23

**Authors:** Jie Liu, Xuan Zou, Yan Zhao, Zhangning Jin, Jun Tu, Xianjia Ning, Jidong Li, Xinyu Yang, Jinghua Wang

**Affiliations:** ^1^Department of Neurology, Tianjin Medical University General Hospital, Tianjin, China; ^2^Laboratory of Epidemiology, Tianjin Neurological Institute, Tianjin, China; ^3^Key Laboratory of Post-Neuroinjury Neuro-repair and Regeneration in Central Nervous System, Ministry of Education, Tianjin Neurological Institute, Tianjin, China; ^4^Center of Clinical Epidemiology & Evidence-Based Medicine, Tianjin Jizhou People's Hospital, Tianjin, China; ^5^Department of Neurosurgery, Tianjin Medical University General Hospital, Tianjin, China; ^6^Department of Neurosurgery, Tianjin Xiqing Hospital, Tianjin, China; ^7^Department of Neurosurgery, Tianjin Jizhou People's Hospital, Tianjin, China

**Keywords:** unruptured intracranial aneurysms, cervical computed tomography angiography, epidemiology, family history, body mass index

## Abstract

Although the prevalence of unruptured intracranial aneurysm (UIA) lies between 2 and 5%, the consequences of aneurysm rupture are fatal. The burden of UIA is considerable in stroke patients. However, the best prevention and management strategy for UIA is uncertain among patients with a family history of stroke. Therefore, this study aimed to determine the epidemiological characteristics and risk factors for UIA based on a population with a family history of stroke. This study used random sampling to recruit participants with a family history of stroke among rural residents in Jixian, Tianjin, China. All participants underwent a questionnaire survey, physical examination, and cervical computed tomography angiography (CTA). CTA data were used to determine whether the subjects had UIA. The relationship between relevant factors and UIA was assessed using logistic regression analysis. A total of 281 residents were recruited in this study, with a mean age of 50.9 years. The prevalence of UIA in those with a family history of stroke was 10.3% overall (9.8% among men and 10.9% among women). Moreover, with each unit increase in body mass index (BMI), the prevalence of UIA decreased by 12.5%. Particularly among non-obese men, BMI had a stronger protective effect (OR: 0.672; 95%CI: 0.499–0.906; *P* = 0.009), and among non-obese men, an increase in low-density lipoprotein (LDL) was associated with an increased prevalence of UIA (OR: 3.638; 95%CI: 1.108–11.947; *P* = 0.033). Among the non-obese with a family history of stroke, BMI may be protective against UIA, especially in men. It is crucial to strictly control the LDL level in non-obese people to reduce the burden of UIA.

## Introduction

Aneurysmal subarachnoid hemorrhage (SAH) is a global health burden and accounts for 5% of all strokes (1–3). The estimated global incidence of SAH is 6.67 per 100,000 people; nearly 500,000 people experience SAH each year, with nearly two-thirds of this population belonging to low- and middle-income countries (4). Aneurysm is only an incidental disease in neurology, with an incidence between 2 and 5% (1, 5, 6). SAH caused by an aneurysm rupture is fatal, with mortality rates of up to 60% (7, 8); even in hospitalized patients, the mortality rate is up to 20% (9). Therefore, it is crucial to promptly detect and provide intervention for unruptured aneurysms, especially in developing countries.

Although aneurysm rupture is the main cause of SAH-type stroke, the impact of stroke on the occurrence and development of aneurysm cannot be ignored. The risk factors for cerebrovascular diseases and cerebral aneurysms are similar (10). Moreover, stroke patients undergoing acute neurovascular examination have a greater vascular burden and higher cerebrovascular sensitivity than the general population (11). Therefore, stroke patients will have a higher burden of unruptured intracranial aneurysms (UIA). At present, studies on UIA have shown that the risk of intracranial aneurysms is 70% higher in stroke patients than in the normal population (12, 13), and a family history of stroke has been identified as an independent risk factor for UIA (12). However, there is no study on the prevalence and risk factors for UIA in those with a family history of stroke in China.

Therefore, this study aimed to determine the epidemiological characteristics and risk factors for UIA based on a population with a family history of stroke and to provide a basis for the prevention and management of UIA among patients with a family history of stroke.

## Methods

### Study Population

The research population was from the Tianjin Brain Research. All participants were from two villages, which were randomly selected among 18 administrative villages. In this study, 75 stroke patients and their immediate family members were recruited from June to August 2013. A questionnaire survey was administered and cerebrovascular computed tomography angiography (CTA) was conducted on all the final recruited subjects. The participants included in this study were all permanent residents aged 18 years and older, with no obvious neurologic impairment (modified Rankin Scale ≤ level 1). Participants with a history of acute myocardial infarction or a mental disorder preventing them from independently answering the questions were excluded (14).

The Ethics Committee of Tianjin Medical University approved this study. Informed consent was obtained from all subjects.

### Questionnaire and Physical Examination

The data for this study were obtained by trained researchers using face-to-face interviews. Demographic information, including sex, age, educational level, and medication history, were captured using predesigned questionnaires. Stroke was considered present if objective imaging evidence could be provided. Cigarette smoking was defined as smoking more than 1 cigarette/day for ≥1 year; participants were categorized as never smokers and smokers. Smoking was defined as a current smoking habit or a previous history of smoking. Drinking was defined as drinking >500 g of alcohol/week for ≥1 year; participants were categorized as never drinkers and drinkers. Drinkers was defined as a current drinking habit or a history of alcohol consumption.

Systolic blood pressure (SBP), diastolic blood pressure (DBP), height, and weight were measured by local general practitioners. Further, fasting levels of blood glucose (FBG), total cholesterol (TC), triglyceride (TG), high-density lipoprotein cholesterol (HDL-C), and low-density lipoprotein cholesterol (LDL-C) were determined at the Tianjin Medical University General Hospital (Tianjin, China). Body mass index (BMI) was calculated as the individual's weight (kg) divided by the square of the height (m^2^); weight classifications were based on BMI (low-weight, <18.5 kg/m^2^; normal, 18.5–23.9 kg/m^2^; overweight, 24.0–27.9 kg/m^2^; and obese, ≥28.0 kg/m^2^) (15). For the multivariate analysis, the obesity group was defined as BMI ≥28.0 kg/m^2^, and the no-obesity group was defined as 18.5 kg/m^2^ ≤ BMI <28.0 kg/m^2^.

### Computed Tomography and Computed Tomography Angiography

This part of the method has been described in detail in previous studies (14). Briefly, participants underwent head CTA. All participants were injected with 60 mL of contrast agent (iodide 370 mg/mL, Bayer, Germany), followed by 40 mL of normal saline collection agent at an injection rate of 4 mL/s. The scan was performed with a 64-section cardiac CT scanner (CT750 HD, GE, USA) using a standardized optimized contrast-enhanced protocol (120 kV [peak]; 180 mAs; collimation, 64 × 0.625 mm; rotation time, 0.5 s; pitch, 1.375). The head CT scan range was from the carotid bifurcation to the parietal region. An aneurysm was defined as an abnormal swelling or bulge of an intracranial artery (16). Aneurysms were jointly diagnosed by a senior radiologist and senior neurosurgeon. None of the patients included in this study had a high suspicion intracranial aneurysm.

### Analysis and Statistics

Age, BMI, SBP, DBP, FBG, TC, TG, HDL-C, and LDL-C were continuous variables and are presented as means and standard deviations; between-group comparisons of these values were performed using Student's *t*-tests. Age, education, BMI, smoking history, drinking history, and previous medical histories were categorical variables and are presented as numbers (n) and frequencies (%); between-group comparisons were performed using the chi-square test. Binary logistic regression was used to evaluate the association between UIA and factors. Blood lipid indexes were tested for collinearity before inclusion in the multivariate analysis. In the multivariate analysis, the independent variables were sex, age, and education in model 1, sex, age, education, hypertension, smoking status, and drinking status in model 2, and sex, age, education, hypertension, smoking status, drinking status, BMI, FBG, and LDL in model 3. The relationships are presented as odds ratios (ORs) with 95% confidence intervals (CIs). All analyses were conducted using SPSS for Windows (version 25.0; SPSS, Chicago, IL, USA). *P* < 0.05 was considered statistically significant.

## Results

### Patient Demographics

**Figure 1:**
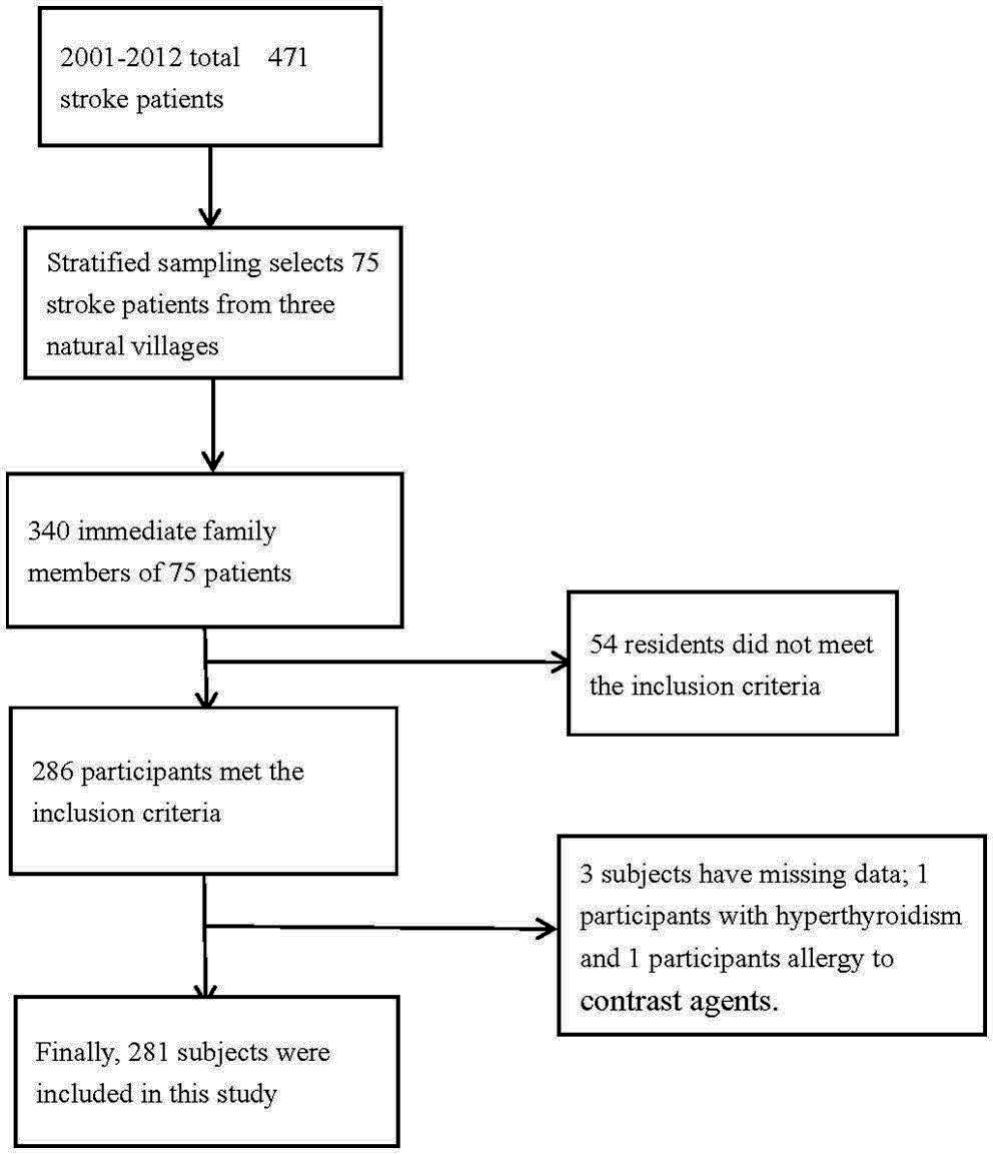
Flow chart of participants' selection.

A total of 340 people with a family history of stroke were enrolled in this study, and 286 participants met the inclusion criteria. However, the basic information of three participants was missing, and two participants were unable to undergo CTA because of hyperthyroidism and allergy to contrast agents. Finally, 281 participants with a family history of stroke were analyzed in this study (Figure 1). The average age of the study population was 50.9 years, and 64.8% of the total population were in the 45–64 age group. The average educational level of this population was low, with an illiteracy rate as high as 10.3%.

Twenty-nine (10.3%) participants demonstrated UIA; the prevalence of UIA was 9.8% and 10.9% in men and women, respectively. The prevalence of hypertension and type II diabetes mellitus was 56.6% and 8.9%, respectively. The average SBP (143.27 mmHg), DBP (89.82 mmHg), FBG (5.14 mmol/L), TC (4.28 mmol/L), TG (1.59 mmol/L), HDL (1.39 mmol/L), and LDL (5.14 mmol/L) levels were also determined (Table 1).

**Table 1:** Demographical and clinical features among people with family history of stroke.

**Characteristics**	**Men**	**Women**	**Total**
Cases, n (%)	153	128	281
Age, year, mean (SD)	50.9 (11.5)	50.8 (9.2)	50.9 (10.5)
Age, n (%)
<45 years	43 (28.1)	33 (25.8)	76 (27.0)
45–64years	93 (60.8)	89 (69.5)	182 (64.8)
≧65years	17 (6.0)	6 (2.1)	23 (8.2)
Education, year, mean (SD)	8 (2.88)	5 (3.94)	7 (3.60)
Education group, n (%)			
0 years	3 (2.0)	26 (20.3)	29 (10.3)
1–6 years	47 (30.7)	44 (34.4)	91 (32.4)
>6 years	103 (67.3)	58 (45.3)	161 (57.3)
BMI, kg/m^2^, mean (SD)	25.67 (3.28)	26.21 (4.91)	25.92 (3.73)
BMI group, n (%)			
Low-weight and normal	47 (30.7)	38 (29.7)	85 (30.3)
Over weight	71 (46.4)	55 (43.0)	126 (44.8)
obesity	35 (22.9)	35 (27.3)	70 (24.9)
SBP, mmHg, mean (SD)	141.67 (22.99)	145.20 (20.32)	143.27 (21.85)
DBP, mmHg, mean (SD)	89.63 (14.86)	90.05 (12.57)	89.82 (13.84)
Medical history, n (%)			
Intracranial aneurysm	15 (9.8)	14 (10.9)	29 (10.3)
Hypertension	79 (51.6)	80 (62.5)	159 (56.6)
Type II DM	15 (9.8)	10 (7.8)	25 (8.9)
Smoking	120 (78.4)	6 (4.7)	126 (44.8)
Drinking	81 (52.9)	5 (3.9)	86 (29.9)
Stroke	10 (6.5)	9 (7.0)	19 (6.8)
Laboratory tests, mean (SD)			
FBG, mmol/L	5.27 (1.09)	4.99 (0.98)	5.14 (1.05)
TC, mmol/L	4.04 (0.73)	4.57 (0.96)	4.28 (0.88)
TG, mmol/L	1.25 (0.61)	1.99 (1.2)	1.59 (0.99)
HDL-C, mmol/L	1.31 (0.42)	1.47 (0.43)	1.39 (0.43)
LDL-C, mmol/L	2.17 (0.65)	2.36 (0.88)	5.14 (1.05)

*SD, standard deviation; SBP, systolic blood pressure; DBP, diastolic blood pressure; BMI, body mass index; FBG, fasting blood glucose; TC, total cholesterol; TG, triglycerides; HDL-C, high density lipoprotein cholesterol; LDL-C, low density lipoprotein cholesterol*.

Moreover, the most common UIA was located in the internal carotid artery, and accounted for 58.6% with the prevalence of 6.04%. There were 96.6% patients with one UIA, with the prevalence of 9.96% (Supplementary Table 1).

### Factors Associated With Unruptured Intracranial Aneurysm in the Univariate Analysis

Although the prevalence of UIA was higher in women than in men (10.9% vs. 9.8%), the difference was not significant (*P* = 0.756). Similarly, the prevalence of UIA was higher in the 45–64 years group, and in those with diabetes, hypertension, and hyperlipidemia, but the differences were not significant (all *P* > 0.05) (Table 2).

**Table 2:** Associated factors of intracranial aneurysm in the univariate analysis among people with family history of stroke.

Category^*^	**No-intracranial**	**Intracranial**	***P***
	**aneurysm**	**aneurysm**	
Total	252 (89.7)	29 (10.3)	
Gender, n (%)			0.756
Men	138 (90.2)	15 (9.8)	
Women	114 (89.1)	14 (10.9)	
Age, means (SD), years	51.02 (10.45)	49.38 (10.67)	0.424
Age group, n (%)			0.583
<45 years	67 (88.2)	9 (11.8)	
45–64years	163 (89.6)	19 (10.4)	
≧65years	22 (95.7)	1 (4.3)	
Education, means (SD), years	6.57 (3.58)	6.79 (3.87)	0.750
Education, n (%)			0.836
0 years	26 (89.7)	3 (10.3)	
1–6 years	83 (91.2)	8 (8.8)	
>6 years	143 (88.8)	18 (11.2)	
Smoking status, n (%)			0.430
Never smoking	137 (88.4)	18 (11.6)	
Smoking	115 (91.3)	11 (8.7)	
Alcohol consumption, n (%)			0.958
Never drinking	175 (89.7)	20 (10.3)	
Drinking	77 (89.5)	9 (10.5)	
Hypertension, n (%)			0.455
No	157 (90.8)	16 (9.2)	
Yes	95 (88.0)	13 (12.0)	
Diabetes, n (%)			0.137
No	234 (89.0)	29 (11.0)	
Yes	18 (100.0)	0	
Hyperlipidemia, n (%)			0.368
No	61 (83.6)	12 (16.4)	
Yes	70 (88.6)	9 (11.4)	
BMI group, n (%)			0.581
Low and normal	74 (87.1)	11 (12.9)	
Overweight	113 (89.7)	13 (10.3)	
Obesity	65 (92.9)	5 (7.1)	
BMI, means (SD), Kg/m^2^	26.04 (3.77)	25.09 (3.68)	0.200
SBP, means (SD), mmHg	143.85 (22.14)	138.28 (18.66)	0.194
DBP, means (SD), mmHg	90.2 (13.90)	86.48 (13.07)	0.171
FBG, means (SD), mmol/L	5.15 (1.08)	5.09 (0.68)	0.795
TC, means (SD), mmol/L	4.28 (0.89)	4.27 (0.84)	0.944
TG, means (SD), mmol/L	1.59 (1.00)	1.53 (0.95)	0.733
HDL-C, means (SD), mmol/L	1.39 (0.43)	1.32 (0.41)	0.394
LDL-C, means (SD), mmol/L	2.25 (0.78)	2.31 (0.67)	0.686

*SD, standard deviation; SBP, systolic blood pressure; DBP, diastolic blood pressure; BMI, body mass index; FBG, fasting blood glucose; TC, total cholesterol; TG, triglycerides; HDL-C, high density lipoprotein cholesterol; LDL-C, low density lipoprotein cholesterol*.

* *Continuous variables were analyzed by variance analysis, and group variables were analyzed by chi-square test*.

### Factors Associated With Unruptured Intracranial Aneurysm in the Multivariate Analysis

In this study, after adjustment for different risk factors, models were established to analyze the relationship between potential risk factors and UIA. When the dependent variable was the presence or absence of aneurysm, there was collinearity among LDL-C, HDL-C, TG, and TC (condition indicator: 49.286). Combined with clinical factors, LDL-C was finally included in multivariate analysis. In model 1 and model 2, no independent risk factors for UIA were identified. However, in model 3, for every unit increase in BMI, the risk of UIA was lowered by 12.5% (95%CI: 0.767–0.998; *P* = 0.047). Age, FBG, LDL, hypertension, smoking status, and drinking status were not significantly related with UIA in this study (all *P* > 0.05) (Table 3).

**Table 3:** Associated factors of intracranial aneurysm in the multivariate analysis among people with family history of stroke.

**Risk factors**	**References**	OR^*^(95% CI)	***P***
Model 1			
Men	Women	0.864 (0.382, 1.953)	0.725
Age	-	0.986 (0.948, 1.026)	0.494
Education	-	1.009 (0.893, 1.141)	0.880
Model 2			
Men	Women	1.114 (0.338, 3.664)	0.860
Age	-	0.985 (0.945, 1.026)	0.456
Education	-	1.001 (0.886, 1.131)	0.986
Hypertension	No		0.426
Yes		1.388 (0.619, 3.113)	
Smoking status	Never smoking		
Smoking		0.641 (0.245, 1.675)	0.364
Drinking status	Never drinking		
Drinking		1.448 (0.522, 4.015)	0.476
Model 3			
Men	Women	1.417 (0.398, 5.042)	0.590
Age	-	0.982 (0.94, 1.025)	0.399
Education	-	0.997 (0.875, 1.136)	0.965
Hypertension	No		
Yes		1.523 (0.614, 3.776)	0.364
Smoking status	Never smoking		
Smoking		0.551 (0.162, 1.878)	0.341
Drinking status	Never drinking		
Drinking		1.436 (0.492, 4.190)	0.507
FBG	-	1.020 (0.68, 1.528)	0.925
LDL	-	1.273 (0.732, 2.215)	0.392
BMI	-	0.875 (0.767, 0.998)	0.047

*Adjusted factors in multivariate analysis: Model 1: adjust gender, age, and education; Model 2: adjust gender, age, education, hypertension, smoking status, and drinking status. Model 3: adjust gender, age, education, hypertension, smoking status, and drinking status, BMI, FBG, and LDL*.

* *Multivariate analysis using binary logistic regression*.

Table 4 shows the relationship between risk factors and UIA in the different BMI groups. In the no-obesity group, for every unit increase in BMI, the risk of UIA was lowered by 18.9% (95% CI: 0.662–0.992; *P* = 0.042), while in the obesity group, there were no significant relationship between BMI and UIA (*P* = 0.851).

**Table 4:** Multivariate analysis the BMI group between BMI and intracranial aneurysms among people with family history of stroke.

**Risk factors**	**References**	OR^*^(95% CI)	***P***
No-obesity group			
Men	Women	2.364 (0.574, 9.732)	0.233
Age	-	0.990 (0.943, 1.039)	0.671
Education	-	1.046 (0.901, 1.214)	0.555
Hypertension	No		
Yes		1.637 (0.573, 4.674)	0.357
Smoking status	Never smoking		
Smoking		0.352 (0.087, 1.425)	0.143
Drinking status	Never drinking		
Drinking		1.822 (0.541, 6.137)	0.333
FBG	-	0.919 (0.573, 1.472)	0.725
LDL	-	1.842 (0.961, 3.527)	0.066
BMI	-	0.811 (0.662, 0.992)	0.042
Obesity group			
Men	Women	0.126 (0.002, 7.643)	0.323
Age	-	0.926 (0.779, 1.099)	0.377
Education	-	0.930 (0.657, 1.317)	0.684
Hypertension	No		
Yes		-	-
Smoking status	Never smoking		
Smoking		1.009 (0.022, 46.124)	0.996
Drinking status	Never drinking		
Drinking		-	-
FBG	-	2.960 (0.566, 15.494)	0.199
LDL	-	0.326 (0.073, 1.463)	0.144
BMI	-	1.042 (0.678, 1.602)	0.851

*Adjust gender, age, education, hypertension, smoking status, and drinking status, BMI, FBG, and LDL*.

* *Multivariate analysis using binary logistic regression*.

To further clarify the relationship between BMI and UIA in the non-obese group, this study conducted a sex-stratified analysis. Table 5 shows that there was a significant relationship between BMI and UIA in men (OR: 0.672; 95%CI: 0.499–0.906; *P* = 0.009). In addition, for every unit increase in LDL, the risk of UIA increased 2.638 times (95%CI: 1.108–11.947; *P* = 0.033). However, in women, no significant associations between BMI, LDL, and UIA were found (OR: 1.047; 95%CI: 0.753–1.457; *P* = 0.784).

**Table 5:** Multivariate analysis the gender differences between BMI and intracranial aneurysms among people with family history of stroke in no-obesity group.

**Risk factors**	**References**	OR^*^(95% CI)	***P***
Men			
Age	-	0.991 (0.931, 1.055)	0.773
Education	-	1.186 (0.911, 1.544)	0.205
Hypertension	No		
Yes		1.963 (0.464, 8.300)	0.359
Smoking status	Never smoking		
Smoking		0.300 (0.058, 1.556)	0.152
Drinking status	Never drinking		
Drinking		3.057 (0.702, 13.322)	0.137
FBG	-	0.753 (0.350, 1.621)	0.469
LDL	-	3.638 (1.108, 11.947)	0.033
BMI	-	0.672 (0.499, 0.906)	0.009
Women			
Age	-	0.965 (0.869, 1.071)	0.499
Education	-	0.941 (0.755, 1.172)	0.586
Hypertension	No		
Yes		1.972 (0.317, 12.27)	0.467
Smoking status	Never smoking		
Smoking		-	-
Drinking status	Never drinking		
Drinking		-	-
FBG	-	1.239 (0.612, 2.508)	0.552
LDL	-	1.080 (0.476, 2.451)	0.854
BMI	-	1.047 (0.753, 1.457)	0.784

*Adjust age, education, hypertension, smoking status, and drinking status, BMI, FBG, and LDL*.

* *Multivariate analysis using binary logistic regression*.

## Discussion

To our knowledge, this is the first study to explore the epidemiological characteristics and factors influencing UIA in low-income people with a family history of stroke in China. In this study, the overall prevalence of UIA in people with a family history of stroke was 10.3% (9.8% among men and 10.9% among women). With each unit increase in BMI, the prevalence of UIA decreased by 12.5% in the overall population and by 32.8% in non-obese men. Moreover, with each unit increase in LDL, the prevalence of UIA increased by 2.638 times in non-obese men.

In recent years, there have been many studies on the prevalence of UIA. The prevalence of intracranial aneurysms was 1.8–3.0% in the general population (1, 5, 17). A previous study reported that the prevalence of UIA was higher among those with transient ischemic attack or minor stroke than in the general population (13), and the prevalence was as high as 5%. However, among the local population in Hong Kong, the prevalence of UIA among residents with immediate family members suffering from aneurysmal SAH was 2.3% (18). Thus, the prevalence of UIA may fluctuate greatly among different populations. In this study, the prevalence of UIA was 10.3%, possibly because of the existence of a genetic susceptibility to aneurysm and other cerebrovascular diseases (11, 19, 20). The study population had a family history of stroke, making the existence of UIA a possibility. Moreover, this population was a low-income and low-education population, which are associated with an extremely high prevalence of stroke (21). Therefore, the above may have caused the high prevalence of UIA in this population.

In this study, there was a significant relationship between BMI and UIA. An increase in BMI was associated with a decreased risk of UIA, especially in men. Similarly, a previous study reported that with decreased BMI, the risk of UIA growth increased (22). Another study reported a positive association between genetically predicted BMI and SAH, while there was no evidence that BMI was related to abdominal or thoracic aortic aneurysm (23). Previous studies of this research cohort showed that the average carotid intima media thickness of the population was low (24). As the average income in this population is low and the diet structure is simple, it may reduce the nutritional status. An increase in BMI at a certain level can better reflect the nutritional status, which may explain why increased BMI reduced the risk of UIA. Moreover, wall stress on the vascular lumen is currently thought to play a key role in the initiation, growth, and rupture of intracranial aneurysms (25). High wall shear stress can promote cell migration and phenotypic changes in smooth muscle cells (SMCs), leading to the secretion of inflammatory mediators and factors involved in the degradation of intracranial aneurysm vessel walls by SMCs (26). This process is thought to be related to the size, growth, and rupture of intracranial aneurysms (27). However, other studies have shown that low BMI is inversely associated with peak wall stress in patients with abdominal aortic aneurysm, leading to an increased risk of rupture of abdominal aortic aneurysms (28, 29). Therefore, patients with relatively high BMI may have lower wall shear stress, thereby reducing the risk of small intracranial aneurysms and rupture. In this study, there is indeed a linear correlation between the BMI and UIA, but a non-linear relationship between BMI and aneurysm cannot be ruled out. The relationship between BMI and aneurysm should be carefully considered as it may help to clarify the mechanism underlying the occurrence and enlargement of aneurysm.

A previous study suggested that aneurysm and atherosclerosis have a common pathological basis (30). Therefore, in this study, we also paid attention to the relationship between blood lipids and aneurysms. This relationship was similar to that obtained in previous research. A study reported that elevated serum LDL-C levels was associated with a 5.8-fold increase in the risk of Takayasu arteritis (31). Another study showed that ruptured aneurysms exhibited significantly higher LDL infiltration than unruptured aneurysms (32). LDL may be involved in the inflammatory process of the arterial intima, and lipid infiltration may promote the occurrence and development of aneurysms. Thus, when assessing the risk of aneurysm enlargement and rupture, the characteristics of lipid infiltration should be considered.

Previous studies have reported risk factors for UIA, including female sex (33), older age (6), hypertension (34), smoking (35), and stroke history (36). In this study, the prevalence of UIA was higher in women and older persons, although these findings were not statistically significant. This may be because the participants in this study were selected from a special population with a family history of stroke, which had a limited sample size. In the future, the research team aims to include a larger sample, with follow-up observation.

There were some limitations in this study. First, this was a small-size single-center study conducted in Tianjin, China. Thus, multicenter, large-size studies are needed to verify the results of the present study. Second, this study was cross-sectional, preventing the determination of a causal link between the risk factors and UIA; thus, a follow-up study involving a similar population is required. Third, aneurysm size was missing in this study, the further study would be needed to deeply assess the detailed status of aneurysm in this population. Finally, this study included a special population. Although the immediate family members did not live together with the participants, the analysis results may have been affected by the similar living habits of the participants and their family members.

## Conclusions

This is the first study to explore the epidemiological characteristics and risk factors for UIA in low-income people with a family history of stroke in rural northern China. The findings suggest that the burden of UIA was notable among people with a family history of stroke. Changes in the BMI of low-income people with a family history of stroke should be seriously considered, especially in men. In non-obese people with a family history of stroke, strict control of LDL and appropriate adjustment of BMI levels may reduce the prevalence of UIA. The nutritional status of this population should also receive further attention.

## Data Availability Statement

The raw data supporting the conclusions of this article will be made available by the authors, without undue reservation.

## Ethics Statement

The studies involving human participants were reviewed and approved by the Ethics Committee of Tianjin Medical University General Hospital. The patients/participants provided their written informed consent to participate in this study.

## Author Contributions

JW, JLi, and XY were involved in conception and design, data interpretation for this article, and critical review in for this article. JLiu, XZ, YZ, ZJ, JT, and XN were involved in data collection, case diagnosis, and confirmation for this article. JLiu, XZ, and YZ were involved in manuscript drafting. JW was involved in data analysis for this article. All authors agree to be accountable for all aspects of the work. All authors contributed to the article and approved the submitted version.

## Conflict of Interest

The authors declare that the research was conducted in the absence of any commercial or financial relationships that could be construed as a potential conflict of interest.

## Publisher's Note

All claims expressed in this article are solely those of the authors and do not necessarily represent those of their affiliated organizations, or those of the publisher, the editors and the reviewers. Any product that may be evaluated in this article, or claim that may be made by its manufacturer, is not guaranteed or endorsed by the publisher.
