# Supplementary material for: Prevalence and Risk Factors for Unruptured Intracranial Aneurysms in the Population at High Risk for Aneurysm in the Rural Areas of Tianjin
Source: Front Neurol. 2022 Mar 23;13:853054. doi: 10.3389/fneur.2022.853054 (PMC8983840; doi:10.3389/fneur.2022.853054)
Supplement: Supplementary file 1 [file Table_1.docx]

Supplementary Table 1. Prevalence of aneurysms at different sites.

| Characteristics | Number | Proportion (%) | Prevalence (%) |
| --- | --- | --- | --- |
| Aneurysm site |  |  |  |
| ICA | 17 | 58.6 | 6.04 |
| ACA | 1 | 3.4 | 0.36 |
| MCA | 1 | 3.4 | 0.36 |
| PCA | 1 | 3.4 | 0.36 |
| ACOA | 2 | 7.0 | 0.71 |
| PCOA | 3 | 10.3 | 1.07 |
| BA | 1 | 3.4 | 0.71 |
| VA | 2 | 7.0 | 0.71 |
| Number of aneurysms: |  |  |  |
| 1 | 28 | 96.6 | 9.96 |
| >1 | 1 | 3.4 | 0.36 |

ICA, internal carotid artery; ACA, [anterior cerebral artery](https://www.baidu.com/link?url=5EwTziGqCgxHk9yyUclvVAnxEuU_I5If6OwSuxKooQEsHEXyvrAdiowvmA-EpGGheQ8XjKys5URc6IVH7VzeVNjphiopZyKnUkOV-u1PSUxSWHV_OLZZSjYnLxENGjkx&wd=&eqid=e9bf1d50000eb03d0000000262176021); MCA, middle cerebral artery; PCA, posterior cerebral artery; ACOA, anterior communicating artery; PCOA, posterior communicating artery; BA, basilar artery; VA, vertebral artery.
